# Silenced SOX2‐OT alleviates ventricular arrhythmia associated with heart failure by inhibiting NLRP3 expression via regulating miR‐2355‐3p

**DOI:** 10.1002/iid3.388

**Published:** 2020-12-03

**Authors:** Yuming Liang, Boqun Wang, Huijuan Huang, Maoyun Wang, Qianwen Wu, Yaxin Zhao, Yan He

**Affiliations:** ^1^ Department of Geriatrics Cardiology the First Affiliated Hospital of Guangxi Medical University Nanning China

**Keywords:** arrhythmias, heart failure, inflammasome, lncRNA, microRNAs

## Abstract

**Background:**

Nucleotide‐binding oligomerization domain‐like receptor family pyrin domain containing 3 (NLRP3) inflammasomes are the most important factors in ventricular arrhythmia associated with heart failure (VA‐HF). However, how the relationship between lncRNA and NLRP3 inflammasomes is regulated in VA‐HF has not been investigated in detail. Thus, we aimed to determine the effects of SOX2‐overlapping transcripts (SOX2‐OT) by targeting NLRP3 in rats with VA‐HF.

**Methods:**

We established rats (SPF, male, weight: 240 ± 10 g) with VA‐HF by aortic coarctation and constant‐current stimulation, then injected them with small interfering SOX2‐OT and anti‐miR‐2355‐3p. Six weeks later, SOX2‐OT and miR‐2355‐3p expression was measured using the quantitative reverse transcriptase‐polymerase chain reaction and NLRP3, ASC, caspase‐1, IL‐1β, and TGF‐β1 expression were measured by Western blot analysis; the ventricular chambers were histopathologically analyzed by staining with hematoxylin and eosin, Masson trichrome, and Picro Sirius Red and reactive oxygen species (ROS) levels were assessed by flow cytometry. The targeting relationship between miR‐2355‐3p and SOX2‐OT or NLRP3 was verified using dual‐luciferase reporter gene assays.

**Results:**

The expression of SOX2‐OT and levels of NLRP3 inflammasomes gradually increased in normal rats, and in those with heart failure and with VA‐HF. Silencing SOX2‐OT expression inhibited NLRP3, ASC, caspase‐1, IL‐1β, and TGF‐β1 expression and ROS production, reduced the degrees of cardiomyocyte necrosis and fibrosis and alleviated cardiac dysfunction in rats with VA‐HF. MicroR‐2355‐3p can bind the SOX2‐OT and the 3′‐untranslated region of NLRP3. Inhibiting miR‐2355‐3p reversed the effect of SOX2‐OT in rats with VA‐HF.

**Conclusions:**

Silencing SOX2‐OT alleviated cardiac dysfunction in rats by reducing the activation of NLRP3 inflammasomes via regulating miR‐2355‐3p.

## Graphical abstract

SOX2‐overlapping transcript (SOX2‐OT) expression and nucleotide‐binding oligomerization domain‐like receptor family pyrin domain containing 3 (NLRP3) inflammasomes are increased in rats with ventricular arrhythmia associated with heart failure (VA‐HF). Silenced SOX2‐OT inhibited NLRP3 inflammasomes, reactive oxygen species levels, and histopathological changes in the myocardia of rats with VA‐HF. Silenced SOX2‐OT alleviates ventricular arrhythmia associated with heart failure by inhibiting NLRP3 expression via regulating miR‐2355‐3p

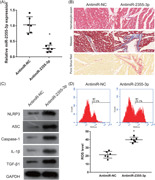

**Abbreviations**
IL: interleukin
NLRP3: nucleotide‐binding oligomerization domain‐like receptor family pyrin domain containing 3
SOX2‐OT: SOX2‐overlapping transcripts
TGF‐β: transforming growth factor‐β
VA‐HF: ventricular arrhythmia associated with heart failure

## 1 BACKGROUND

Chronic heart failure (HF) is the end‐stage manifestation of cardiovascular disease, which is mainly caused by hypertension, cardiomyopathy, as well as coronary and valvular heart diseases.^1^ The rate of sudden death among patients with HF is six‐ to ninefold that of the general population, and 50%–70% of sudden cardiac deaths are due to malignant ventricular arrhythmias (VA) including tachycardia and fibrillation.^2^, ^3^ The main cause of death among patients with heart disease is VA, which needs to be resolved through appropriate investigation.

Inflammation is the most important factor in the induction of cardiomyocyte necrosis, because, with subsequent electrical and structural remodeling, it leads to VA. C‐reactive protein, interleukin (IL)‐1β, IL‐2, IL‐6, IL‐8, and tissue necrosis factor (TNF‐α), are inflammatory markers that become increased in VA, and C‐reactive protein, IL‐6, and white blood cells are independently associated with the incidence of HF.^4^, ^5^, ^6^ Transforming growth factor‐β (TGF‐β) promotes extracellular matrix remodeling and cardiac collagen expression that contribute to diastolic dysfunction.^7^ IL‐1β aggravates VA induced by ischemia.^8^ Inhibiting the expression of TGF‐β, IL‐1β, IL‐6, IL‐18, and TNF‐α can reduce VA in ischemic HF, which plays a role in anti‐VA.^9^, ^10^, ^11^ Nucleotide‐binding oligomerization domain‐like receptor family pyrin domain containing 3 (NLRP3) inflammasomes were recently discovered as inflammatory response regulators that consist of NLRP3, ASC, and caspase‐1. Activating NLRP3 inflammasomes can promote the release of IL‐1β and IL‐18, which triggers a cascade of downstream inflammation that eventually leads to local or systemic inflammation.^12^ Abnormal activation of NLRP3 inflammasomes can lead to cardiac inflammation and heart dysfunction.^13^, ^14^ The activation of NLRP3 inflammasomes increases IL‐1β release to promote myocardial inflammation, systolic dysfunction, and VA.^15^, ^16^ Additionally, silencing NLRP3 inflammasomes can reduce inflammation and improve cardiac dysfunction.^17^ These findings show that NLRP3 inflammasomes comprise a novel mediator in patients with ventricular arrhythmia associated with heart failure (VA‐HF). However, the regulatory mechanism of NLRP3 inflammasome activation in VA‐HF remains unknown.

Long noncoding ribonucleic acids (lncRNA) play important functional roles in cardiovascular diseases. The lncRNA LIPCAR is a biomarker of cardiac remodeling that also independently predicts the death of patients with HF.^18^ The expression of lncRNA Heat2 is significantly increased in the blood of patients with HF, and it regulates inflammatory and immune functions.^19^ Downregulated lncRNA GASL1 promotes cardiomyocyte apoptosis via TGF‐β1 activation.^20^ The lncRNA SOX2‐overlapping transcripts (SOX2‐OT), is transcribed in the same orientation as an SRY‐box containing gene 2 that is embedded in an intron of the SOX2‐OT gene. The expression of SOX2‐OT is significantly increased in HF.^21^ However, the expression and functions of SOX2‐OT in VA‐HF remain unclear.

We constructed a rat model of VA‐HF to determine SOX2‐OT expression as well as the mechanism through which silencing SOX2‐OT improves heart failure by inhibiting NLRP3 expression.

## 2 METHODS

All animal experiments were performed in accordance with the National Institutes of Health Guidelines on the Use of Laboratory Animals and were approved by the Animal Care and Welfare Committee at Guangxi Medical University (Approval No. 201711060).

### 2.1 Model induction and experimental design

SPF Male Sprague–Dawley rats (*n* = 48; weight, 240 ± 10 g) purchased from Experimental animal center of Guangxi Medical University (Nanning City, China) were housed at 23°C ± 2°C with 55% ± 5% humidity and 12:12 h equivalent day–night cycles. The rats had free access to food and water. After adaptation feeding for 1 week, the rats were randomly assigned to sham (*n* = 6) and HF (*n* = 40) groups. We established models of HF by aortic coarctation as described.^22^ Briefly, after shaving the left side of the chest, the rats were anesthetized by 3% pentobarbital sodium (30 mg/kg intraperitoneally) inhalation. Heart failure was established by surgical aortic coarctation, then the rats were administered with penicillin (100,000 IU/d intramuscularly) for 3 days. Chronic heart failure was complete in the models after 4 weeks. The sham group underwent a similar surgical procedure without aortic coarctation. The HF model was stimulated with a 0.8 mA constant‐current for 2 ms in the left cervical sympathetic ganglion to construct a VA model. Rats that developed ventricular tachycardia and fibrillation were assigned to a VA‐HF group (*n* = 32), whereas those that did not were assigned to a VA‐HF control group (*n* = 8). The VA‐HF model rats were randomly assigned to VA‐HF, small interfering negative control (si‐NC), si‐SOX2‐OT, si‐SOX2‐OT + negative control sequence (anti‐miR‐NC), and si‐SOX2‐OT + anti‐miR‐miR‐2355‐3p groups (*n* = 6 per group). Each group was processed as follows: 100 μg si‐NC, si‐SOX2‐OT, si‐SOX2‐OT and anti‐miR‐NC, and si‐SOX2‐OT and anti‐miR‐2355‐3p were mixed with 50 μl of Entranster™—in vivo (animal in vivo transfection reagent, specially used for animals in vivo transfection, can directly transfer RNA into the animal body, No. 18668‐11‐1, Engreen Biosystem Co., Ltd.), and injected into the tail veins of the rats every 3 days for 4 weeks.^22^ All animals were housed in the same room and the treatments were carried out in the order before grouping. Only therapists aware of the group allocation at the different stages of the experiment. All animals were killed at 6 weeks thereafter by an overdose of pentobarbital sodium (120 mg/kg). The following characteristics were considered humane endpoints to the study that required immediate intervention: Infection at the surgical site, rapid weight loss (>20% body weight loss), becoming cachectic, self‐harming, biting or aggressive, and difficulty eating, drinking, or moving around freely.

### 2.2 Tissue processing

The ventricular chamber was flushed with 0.9% sodium chloride chilled at 4°C to remove blood. Myocardial tissues were immediately cut into 2 to 3‐mm wide sections and fixed in 4% paraformaldehyde for 4 h for hematoxylin‐eosin staining (H&E staining), Masson staining, Picro Sirius Red staining, and apoptotic cell detection. Portions of the tissues were placed in cryotubes and stored under liquid nitrogen for subsequent determination of messenger RNA and related protein expression.

### 2.3 Reverse transcription‐quantitative polymerase chain reaction (qRT‐PCR)

The expression of SOX2‐OT and miR‐2355‐3p was measured by qRT‐PCR. Total RNA extracted using Trizol (Invitrogen), was reverse‐transcribed to obtain cDNA according to the ImProm‐IITM Reverse Transcription System (Promega Corp.). Quantitative PCR proceeded using SYBR GREEN qPCR Super Mix (Invitrogen), and expression levels were calculated using the $2^{‐{\mathrm{\Delta \Delta C}}_{t}}$ method. The experiment was repeated three times and the final data were averaged. The designed forward and reverse primers for SOX2‐OT, miR‐2355‐3p, β‐actin, and U6 (Genewiz) were as follows respectively: SOX2‐OT: 5′‐CAACTCGTTCTGTCCGGTGA‐3′ and 5′‐CCATGCCAGAGCAGGGTGTT‐3′; β‐actin: 5′‐CACCCGCGAGTACAACCTTC‐3′ and 5′‐CCCATACCCACCATCACACC‐3′; miR‐2355‐3p: 5′‐ACACTCCAGCTGGGCAAAGAATTCTCCTTT‐3′ and 5′‐CTCAACTGGTGTCGTGGA‐3′; U6: 5′‐CTCGCTTCGGCAGCACA3′ and 5′‐AACGCTTCACGAATTTGCGT3′.

### 2.4 Staining with H&E, Masson trichrome, and Picro Sirius Red

All outcome measures assessed according to the pathology results of H&E, Masson trichrome, and Picro Sirius Red. The ventricular chambers were washed with PBS for 30 min, dehydrated in a graded series of 50%, 70%, 90%, and 100% ethanol for 2 h each, cleared in xylene overnight, then infiltrated with paraffin. Tissue blocks were trimmed and sliced into 5‐µm sections that were stained with H&E, (ab245880, Abcam plc.) Masson trichrome (ab150669, Abcam plc.), and Picro Sirius Red (ab150681, Abcam plc.) using respective kits as described by the manufacturer. Cardiomyocyte cross‐sectional areas, changes in myocardial fibrosis, and the distribution of collagen I were assessed in 10 fields per sample by light microscopy (Olympus) and analyzed using Image‐Pro Plus 6.0 software (Media Cybernetics, Inc.). Masson trichrome stained collagen fibers and cardiomyocytes blue and red, respectively, and Picro Sirius Red stained collagen I red.

### 2.5 Western blot analysis

The expression of NLRP3, ASC, caspase‐1, IL‐1β, and TGF‐β1 was measured by Western blot analysis. Total proteins in ventricular chamber tissues were isolated using radioimmunoprecipitation assay buffer, and concentrations were measured using BCA Protein Assay Kits (Beyotime Institute Biotechnology). Total protein was separated by 10% polyacrylamide gel electrophoresis and transferred onto polyvinylidene fluoride membranes. Nonspecific binding was blocked with 5% skim milk in Tris‐buffered saline Tween‐20, then the membranes were incubated for 4 h at 37°C with primary antibodies against NLRP3 (ab263899, 1:1000), ASC (ab175449, 1 μg/ml), caspase‐1 (ab207802, 1:1000), IL‐1β (ab9722, 0.1 μg/ml), TGF‐β1 (ab179695, 1:1000), and glyceraldehyde 3‐phosphate dehydrogenase (GAPDH) (ab181602, 1:10000) (Abcam), followed by the corresponding secondary antibodies (ab205718/ab205723, Abcam) for 1 h. The membranes were rinsed, then proteins were visualized using enhanced chemiluminescence. Relative protein expression was calculated as the ratio of target protein expression to that of the reference protein (GAPDH). The experiment was repeated three times and the data were averaged.

### 2.6 ROS assays

The generation ROS was measured using a dihydroethidium (DHE) probe (Beyotime). Briefly, cardiomyocytes were separated and incubated with the DHE (50 μM) probe for 30 min at 37°C. Levels of ROS were analyzed using a FACSCalibur flow cytometry system (Becton Dickinson & Co., D) with an argon‐ion laser tuned to 485 nm.

### 2.7 Dual‐luciferase reporter gene assays

MicroRNA (miRNA) sponged by SOX2‐OT were predicted using StarBase 3.0,^23^ and miRNA that regulate the 3′‐untranslated region (UTR) of NLRP3 were predicted using TargetScan (http://www.targetscan.org/). Wild‐type (WT) or mutant SOX2‐OT and the WT or mutant 3′‐UTR of NLRP3 were cloned into the 3′‐end of the firefly luciferase gene of the psi‐CHECK2 vector. Thereafter, 30 ng of either WT or mutant constructs plasmid were cotransfected with 50 nM of either miR‐2355‐3p mimics or negative control into HEK 293 T cells derived from the HEK 293 cell line that expresses a mutant version of the SV40 large T antigen. Luciferase activity was measured 48 h later using a dual‐luciferase assay kit (Promega). The activity of *Renilla* luciferase was normalized against that of firefly luciferase.

### 2.8 Statistical analysis

All data are shown as means ± standard deviation and were statistically analyzed using SPSS 19.0 software (IBM Corp.). Differences among the six groups which are normally distributed data were evaluated using one‐way analyses of variance, followed by Tukey post‐hoc tests. Nonnormally distributed count data were analyzed using the rank‐sum test. Values with *p* < .05 were considered statistically significant.

## 3 RESULTS

### 3.1 SOX2‐OT expression and NLRP3 inflammasomes are increased in rats with VA‐HF

**Figure 1:**
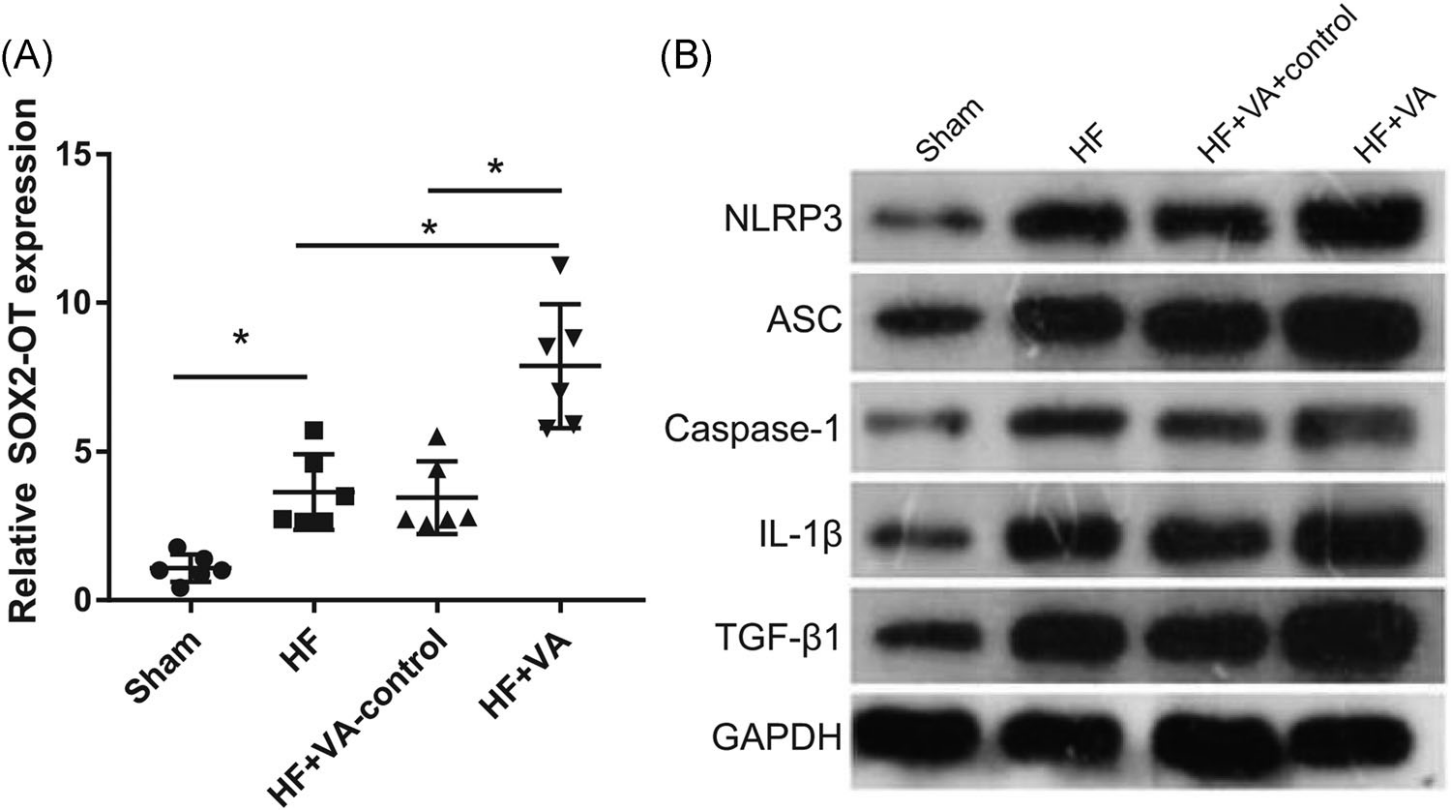
Expression of SOX2‐OT and NLRP3 is increased in rats with VA‐HF. (A) SOX2‐OT expression measured by qRT‐PCR. (B) NLRP3, ASC, caspase‐1, IL‐1β, and TGF‐β1 expression measured by Western blot analysis. GAPDH, glyceraldehyde 3‐phosphate dehydrogenase; HF, heart failure; IL‐1β, interleukin‐1β; NLRP3, nucleotide‐binding oligomerization domain‐like receptor family pyrin domain containing 3; qRT‐PCR, quantitative reverse transcriptase‐polymerase chain reaction; SOX2‐OT, SOX2‐overlapping transcripts; TGF‐β1, transforming growth factor‐β1; VA, ventricular arrhythmia. *p < .05 (*n* = 6 per group)

The results showed the expression of SOX2‐OT was significantly higher in the HF than the sham group, and further enhanced compared with the HF and VA‐HF‐control groups (Figure 1A). We also found that NLRP3, ASC, caspase‐1, IL‐1β, and TGF‐β1 expression was significantly higher in the HF than in the sham group. Compared with the HF and VA‐HF‐control groups, the expression of NLRP3, ASC, caspase‐1, IL‐1β, and TGF‐β1 was further enhanced in the VA‐HF group (Figure 1B).

### 3.2 Silenced SOX2‐OT inhibited NLRP3 inflammasomes, ROS levels, and histopathological changes in the myocardia of rats with VA‐HF

**Figure 2:**
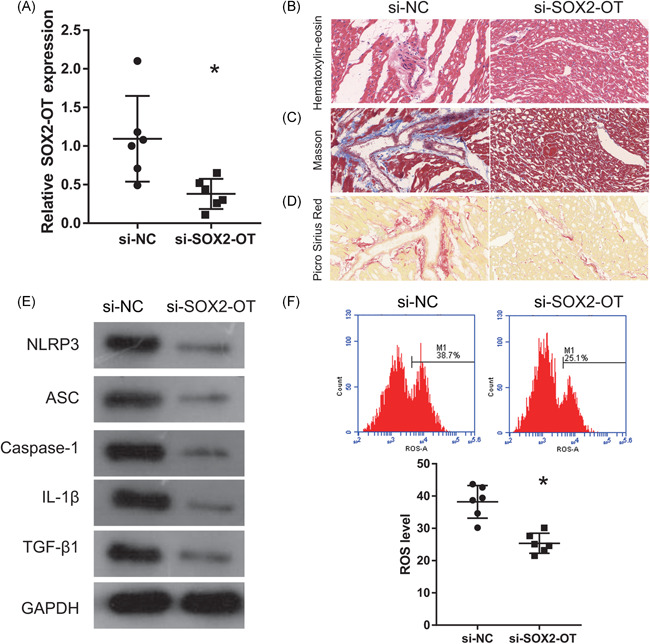
Silenced SOX2‐OT expression inhibits NLRP3 inflammasomes, reduces ROS levels and myocardial histopathological changes in rats V with VA‐HF. (A) Expression of SOX2‐OT measured by qRT‐PCR 6 weeks after injection of si‐SOX2‐OT. (B–D) Ventricular chambers stained with H&E, Masson trichrome, and Picro Sirius Red 6 weeks after injection of si‐SOX2‐OT (200×). (E) Caspase‐1, NLRP3, ASC, IL‐ 1β, and TGF‐β1 expression measured by western blot analysis 6 weeks after injection of si‐SOX2‐OT. (F) Levels of ROS measured by flow cytometry 6 weeks after injection of si‐SOX2‐OT. GAPDH, glyceraldehyde 3‐phosphate dehydrogenase; H&E, hematoxylin‐eosin; IL‐1β, interleukin‐1β; NLRP3, nucleotide‐binding oligomerization domain‐like receptor family pyrin domain containing 3; qRT‐PCR, quantitative reverse transcriptase‐polymerase chain reaction; ROS, reactive oxygen species; si‐NC, small interfering negative control; SOX2‐OT, SOX2‐overlapping transcripts; TGF‐β1, transforming growth factor‐β1; VA‐HF, ventricular arrhythmia associated with heart failure. *p < .05 (*n* = 6 per group)

We silenced SOX2‐OT expression to determine its function in rats with VA‐HF. The expression of SOX2‐OT was significantly reduced in the ventricular chambers after rats were injected with si‐SOX2‐OT (Figure 2A). Staining with H&E showed swollen and necrotic cardiomyocytes in the VA‐HF group, an edematous myocardial cell gap, cytoplasmic vacuolization, and broken, dissolved, and disordered myocardial fibers in the si‐NC group. In contrast, the number of cardiomyocytes was basically normal, muscle fibers were properly arranged, and the degree of necrosis among cardiomyocytes was relatively mild after injecting si‐SOX2‐OT into rats with VA‐HF (Figure 2B). Masson and Picro Sirius Red staining revealed obviously increased fibrosis in the si‐NC group that was improved after injecting si‐SOX2‐OT into rats with VA‐HF (Figure 2C,D). The expression of NLRP3, ASC, caspase‐1, IL‐1β, and TGF‐β1 and the ROS levels were obviously inhibited in the si‐SOX2‐OT, compared with the si‐NC group (Figure 2E,F).

### 3.3 miR‐2355‐3p is a potential target miRNA of SOX2‐OT

**Figure 3:**
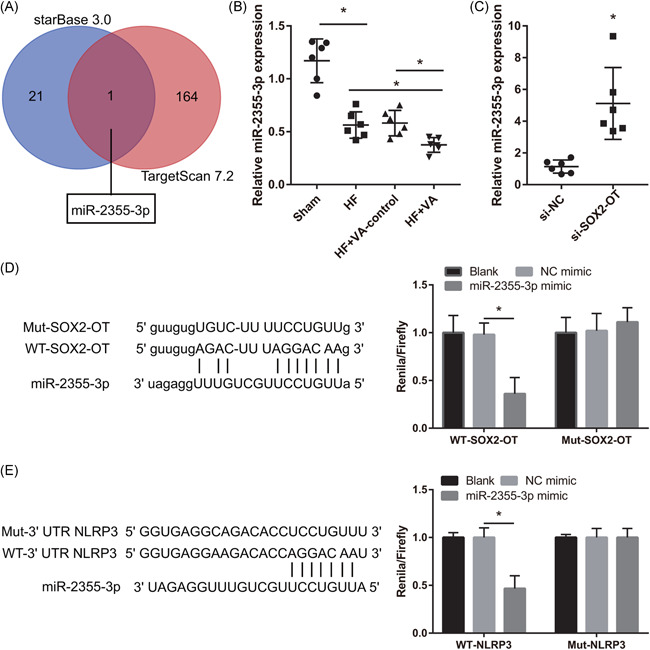
MicroR‐2355‐3p is a potential target miRNA (miR) of SOX2‐OT. (A) MicroRNA binding with SOX2‐OT and the 3′‐UTR of NLRP3 in miR‐2355‐3p was analyzed using starBase 3.0 and target 7.2. (B) Expression of miR‐2355‐3p measured by qRT‐PCR in sham, HF, VA‐HF‐control, and VA‐HF groups. (C) Expression miR‐2355‐3p measured by qRT‐PCR in si‐NC and si‐SOX2‐OT at 6 weeks after injection in rats with VA‐HF. (D) Binding between miR‐2355‐3p and SOX2‐OT. (E) Binding between miR‐2355‐3p and 3′‐UTR NLRP3 analyzed by dual‐luciferase reporter gene assays. GAPDH, glyceraldehyde 3‐phosphate dehydrogenase; HF, heart failure; IL‐1β, interleukin‐1β; Mut, mutant; NLRP3, nucleotide‐binding oligomerization domain‐like receptor family pyrin domain containing 3; qRT‐PCR, quantitative reverse transcriptase‐polymerase chain reaction; ROS, reactive oxygen species; si‐NC, small interfering negative control; SOX2‐OT, SOX2‐overlapping transcripts; TGF‐β1, transforming growth factor‐β1; UTR, untranslated region; VA, ventricular arrhythmia; WT, wild‐type. *p < .05

MicroRNAs (miRNAs) binding to SOX2‐OT and the 3′‐UTR of NLRP3 in miR‐2355‐3p was analyzed using starBase 3.0 and target 7.2 (Figure 3A). The results of qRT‐PCR results showed significantly lower miR‐2355‐3p expression in rats with HF than sham rats, and that this was further reduced in the VA‐HF, than in the HF and VA‐HF‐control groups (Figure 3B). Furthermore, miR‐2355‐3p expression was significantly increased in ventricular chambers after si‐SOX2‐OT injections (Figure 3C). *Renilla*/firefly luciferase activities were significantly lower in co‐miR‐2355‐3p mimic and WT‐SOX2‐OT or WT‐3′‐UTR NLRP3 groups than in co‐NC mimic and WT‐SOX2‐OT or WT‐3′‐UTR NLRP3 groups. *Renilla*/firefly luciferase activity was essentially unchanged in the co‐miR‐2355‐3p mimic and mut‐SOX2‐OT or mut‐WT‐3′‐UTR NLRP3 groups compared with the co‐NC mimic and mut‐SOX2‐OT or mut‐3′‐UTR NLRP3 groups (Figure 3D,E).

### 3.4 miR‐2355‐3p reverses the effect of SOX2‐OT in VA‐HF rats

**Figure 4:**
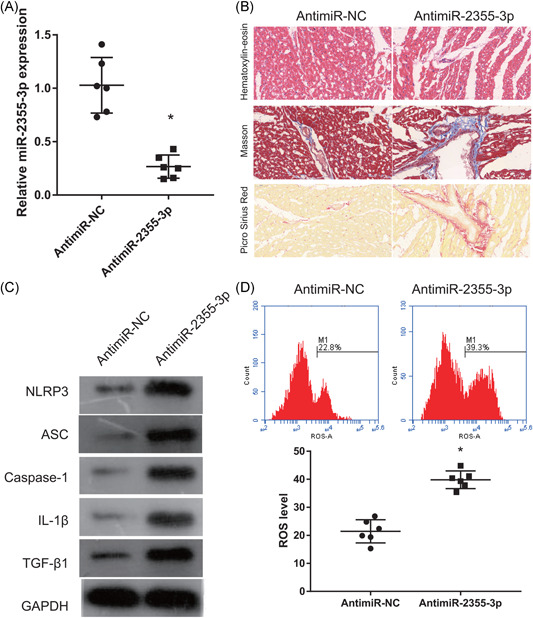
MicroR‐2355‐3p reverses SOX2‐OT effects in rats with VA‐HF. (A) SOX2‐OT expression measured by qRT‐PCR 6 weeks after coinjection of si‐SOX2‐OT and anti‐miR‐2355‐3p. (B) Ventricular chambers stained with H&E, Masson trichrome, and Picro Sirius Red 6 weeks after coinjection of si‐SOX2‐OT and anti‐miR‐2355‐3p (200×). (C) Caspase‐1, NLRP3, ASC, IL‐1β, and TGF‐β1 expression measured by Western blot analysis 6 weeks after coinjection of si‐SOX2‐OT and anti‐miR‐2355‐3p. (D) Levels of ROS measured by flow cytometry 6 weeks after coinjection of si‐SOX2‐OT and anti‐miR‐2355‐3p. Anti‐miR‐NC group: coinjected with si‐SOX2‐OT and anti‐miR‐2355‐3p negative control; anti‐miR‐2355‐3p group: coinjected with si‐SOX2‐OT and anti‐miR‐2355‐3p. GAPDH, glyceraldehyde 3‐phosphate dehydrogenase; HF, heart failure; H&E, hematoxylin‐eosin; IL‐1β, interleukin‐1β; NLRP3, nucleotide‐binding oligomerization domain‐like receptor family pyrin domain containing 3; miR, microRNA; qRT‐PCR, quantitative reverse transcriptase‐polymerase chain reaction; ROS, reactive oxygen species; si, small interfering; SOX2‐OT, SOX2‐overlapping transcripts. *p < .05 (*n* = 6 per group)

We investigated the effects of miR‐2355‐3p on SOX2‐OT by coinjecting si‐SOX2‐OT and anti‐miR‐2355‐3p into rats with VA‐HF. We found significantly reduced miR‐2355‐3p expression in the ventricular chambers of rats coinjected with si‐SOX2‐OT and anti‐miR‐2355‐3p than with si‐SOX2‐OT and anti‐miR‐NC (Figure 4A). Staining with H&E, Masson trichrome, and Picro Sirius Red showed swollen, necrotic cardiomyocytes, broken, dissolved, and disorderly myocardial fibers, and obviously increased fibrosis in rats with VA‐HF that were coinjected with si‐SOX2‐OT and anti‐miR‐2355‐3p than with si‐SOX2‐OT and anti‐miR‐NC (Figure 4B). Caspase‐1, NLRP3, ASC, IL‐1β, and TGF‐β1 expression, as well as ROS levels, were obviously increased in rats coinjected with si‐SOX2‐OT and anti‐miR‐2355‐3p compared with si‐SOX2‐OT and anti‐miR‐NC (Figures 4C,D).

## 4 DISCUSSION

Ventricular arrhythmia associated with heart failure is involved in high morbidity and mortality rates.^24^ Here, we found increased SOX2‐OT expression and NLRP3 inflammasome levels in rats with VA‐HF. Silencing SOX2‐OT reduced ROS levels and the numbers of NLRP3 inflammasomes and alleviated histopathological lesions and cardiomyocyte fibrosis in rats with VA‐HF. We also found that miR‐2355‐3p, which is a target of SOX2‐OT, can reverse the effect of SOX2‐OT in rats with VA‐HF.

The regulatory role played by SOX2‐OT in human diseases including bipolar disorder and schizophrenia is important.^25^, ^26^ Furthermore, SOX2‐OT can predict poor overall and disease‐free survival, and correlates with tumor development and metastasis; thus, it serves as a novel diagnostic marker and plays oncogenic roles in many cancers.^27^, ^28^ The expression of SOX2‐OT gradually increases in healthy persons, and in patients with end‐, and non‐end‐stage HF.^29^ We found that SOX2‐OT expression gradually increased in rats that were healthy, and in those with HF and VA‐HF. The findings were similar to those of a previous study. Additionally, silenced SOX2‐OT expression reduced the degree of cardiomyocyte necrosis and fibrosis and alleviated cardiac dysfunction in rats with VA‐HF.

A major new finding of this study is that silencing SOX2‐OT inhibited NLRP3, ASC, caspase‐1, IL‐1β, and TGF‐β1 expression. The inflammatory markers, IL‐1β and TGF‐β1 promote extracellular matrix remodeling and cardiac collagen expression and aggravate VA induced by ischemia.^4^, ^7^, ^8^ Caspase‐1 NLRP3 and ASC are components of NLRP3 inflammasomes that can cause cardiac inflammation and cardiac dysfunction via IL‐1β and TGF‐β1 release.^13^, ^14^, ^15^, ^16^ Inhibiting TGF‐β and IL‐1β expression can alleviate cardiac dysfunction in ischemic VA‐HF.^9^, ^10^, ^11^ The present study found that NLRP3 inflammasomes and the expression of IL‐1β and TGF‐β1 gradually increased in healthy rats, and in rats with HF and VA‐HF. These findings were in line with previous results. The overproduction of ROS is a key influencing factor that can induce heart failure, cardiac arrhythmias, and other pathological states.^30^ Levels of ROS are significantly increased in myocytes incubated with IL‐1β and TNF‐α.^31^ We found here that silencing SOX2‐OT reduced ROS levels that were increased in rats with VA‐HF. These results showed that SOX2‐OT can regulate the NLRP3 inflammasome/IL‐1β/ROS signaling pathway.

The relationship between lncRNA and NLRP3 has been discussed. The lncRNA MEG3/miR‐223/NLRP3 axis in atherosclerosis promotes endothelial cell pyroptosis.^32^ The lncRNA MALAT1/miR‐22/NLRP3 promotes high levels of glucose‐induced human endothelial cells pyroptosis.^33^ The LINC00339/miR‐22‐3p/NLRP3 axis in kidney stones induced by calcium oxalate promotes renal tubular epithelial pyroptosis.^34^ These results showed that lncRNA regulate NLRP3 expression by sponging miRNA. Others have also found that SOX2‐OT can sponge miRNA such as miR‐654 and miR‐369‐3p and that miR‐146b‐5p facilitates cancer cell proliferation and migration.^35^, ^36^, ^37^ Here, we found that the target miRNA of SOX2‐OT and NLRP3 is miR‐2355‐3p. The expression of SOX2‐OT gradually decreased in healthy rats, and in rats with HF and VA‐HF. We also found that miR‐2355‐3p reversed the effects of SOX2‐OT on the degree of cardiomyocyte necrosis and fibrosis, the expression of NLRP3, ASC, caspase‐1, IL‐1β, and TGF‐β1, and levels of ROS. These results suggested that silencing SOX2‐OT inhibited NLRP3 expression by sponging miR‐2355‐3p, then inhibiting IL‐1β and TGF‐β1 expression and reducing ROS levels, resulting in alleviated cardiac dysfunction in rats with VA‐HF.

## 5 CONCLUSIONS

The present findings revealed that silencing SOX2‐OT alleviated cardiac dysfunction by reducing the activation of NLRP3 inflammasomes. MicroR‐93 might serve as a novel therapeutic target for the prevention of VA‐HF.

## CONFLICT OF INTERESTS

The authors declare that there are no conflict of interests.

## AUTHOR CONTRIBUTIONS

Yuming Liang and Yan He have made contributions to the conception and design of the work. Yuming Liang, Boqun Wang, and Huijuan Huang analyzed and interpreted the data. Yuming Liang, Maoyun Wang, Qianwen Wu, and Yaxin Zhao prepared and assessed histopathological sections of the heart and contributed to revising the manuscript. Yuming Liang drafted the work and Yan He has substantively revised it.

## Data Availability

All data generated or analyzed during this study are included in this published article.

## References

[1] Buckley LF , Shah AM . Recent advances in the treatment of chronic heart failure. F1000Res. 2019;8:8. 31942237 10.12688/f1000research.20447.1 PMC6944255

[2] Goldberger JJ , Buxton AE , Cain M , et al. Risk stratification for arrhythmic sudden cardiac death: identifying the roadblocks. Circulation. 2011;123:2423‐2430. 21632516 10.1161/CIRCULATIONAHA.110.959734

[3] Alenazy B , Tharkar S , Kashour T , Alhabib KF , Alfaleh H , Hersi A. In‐hospital ventricular arrhythmia in heart failure patients: 7‐year follow‐up of the multi‐centric HEARTS registry. ESC. Heart Fail. 2019;6:1283‐1290. 10.1002/ehf2.12525 PMC6989287 31750631

[4] Lewek J , Kaczmarek K , Cygankiewicz I , Wranicz JK , Ptaszynski P. Inflammation and arrhythmias: potential mechanisms and clinical implications. Expert Rev Cardiovasc Ther. 2014;12:1077‐1085. 25060800 10.1586/14779072.2014.942286

[5] Eisen A , Benderly M , Behar S , Goldbourt U , Haim M. Inflammation and future risk of symptomatic heart failure in patients with stable coronary artery disease. Am Heart J. 2014;167:707‐714. 24766981 10.1016/j.ahj.2014.01.008

[6] Safranow K , Dziedziejko V , Rzeuski R , et al. Inflammation markers are associated with metabolic syndrome and ventricular arrhythmia in patients with coronary artery disease. Postepy Hig Med Dosw (Online). 2016;70:56‐66. 26864064 10.5604/17322693.1194612

[7] Westermann D , Lindner D , Kasner M , et al. Cardiac inflammation contributes to changes in the extracellular matrix in patients with heart failure and normal ejection fraction. Circ Heart Fail. 2011;4:44‐52. 21075869 10.1161/CIRCHEARTFAILURE.109.931451

[8] Wang M , Li S , Zhou X , et al. Increased inflammation promotes ventricular arrhythmia through aggravating left stellate ganglion remodeling in a canine ischemia model. Int J Cardiol. 2017;248:286‐293. 28826800 10.1016/j.ijcard.2017.08.011

[9] Wu S , Lin Z , Lin Y , et al. Dexmedetomidine exerted anti‐arrhythmic effects in rat with ischemic cardiomyopathy via upregulation of connexin 43 and reduction of fibrosis and inflammation. Front Physiol. 2020;11:33. 32116751 10.3389/fphys.2020.00033 PMC7020758

[10] Chang SL , Hsiao YW , Tsai YN , et al. Interleukin‐17 enhances cardiac ventricular remodeling via activating MAPK pathway in ischemic heart failure. J Mol Cell Cardiol. 2018;122:69‐79. 30096409 10.1016/j.yjmcc.2018.08.005

[11] Wang S , Wu L , Li X , et al. Light‐emitting diode therapy protects against ventricular arrhythmias by neuro‐immune modulation in myocardial ischemia and reperfusion rat model. J Neuroinflammation. 2019;16:139. 31287006 10.1186/s12974-019-1513-5 PMC6615251

[12] Schroder K , Tschopp J. The inflammasomes. Cell. 2010;140:821‐832. 20303873 10.1016/j.cell.2010.01.040

[13] Luo B , Wang F , Li B , et al. Association of nucleotide‐binding oligomerization domain‐like receptor 3 inflammasome and adverse clinical outcomes in patients with idiopathic dilated cardiomyopathy. Clin Chem Lab Med. 2013;51:1521‐1528. 23382313 10.1515/cclm-2012-0600

[14] Kobayashi M , Usui‐Kawanishi F , Karasawa T , et al. The cardiac glycoside ouabain activates NLRP3 inflammasomes and promotes cardiac inflammation and dysfunction. PLOS One. 2017;12:e0176676. 28493895 10.1371/journal.pone.0176676 PMC5426608

[15] Yang HJ , Kong B , Shuai W , Zhang JJ , Huang H. Knockout of MD1 contributes to sympathetic hyperactivity and exacerbates ventricular arrhythmias following heart failure with preserved ejection fraction via NLRP3 inflammasome activation. Exp Physiol. 2020;105:966‐978. 32240565 10.1113/EP088390

[16] Bracey NA , Beck PL , Muruve DA , et al. The Nlrp3 inflammasome promotes myocardial dysfunction in structural cardiomyopathy through interleukin‐1beta. Exp Physiol. 2013;98:462‐472. 22848083 10.1113/expphysiol.2012.068338

[17] Takahashi M. Role of NLRP3 inflammasome in cardiac inflammation and remodeling after myocardial infarction. Biol Pharm Bull. 2019;42:518‐523. 30930410 10.1248/bpb.b18-00369

[18] Kumarswamy R , Bauters C , Volkmann I , et al. Circulating long noncoding RNA, LIPCAR, predicts survival in patients with heart failure. Circ Res. 2014;114:1569‐1575. 24663402 10.1161/CIRCRESAHA.114.303915

[19] Boeckel JN , Perret MF , Glaser SF , et al. Identification and regulation of the long non‐coding RNA Heat2 in heart failure. J Mol Cell Cardiol. 2019;126:13‐22. 30445017 10.1016/j.yjmcc.2018.11.004

[20] Deng H , Ouyang W , Zhang L , Xiao X , Huang Z , Zhu W. LncRNA GASL1 is downregulated in chronic heart failure and regulates cardiomyocyte apoptosis. Cell Mol Biol Lett. 2019;24:41. 31223316 10.1186/s11658-019-0165-x PMC6567419

[21] Greco S , Zaccagnini G , Perfetti A , et al. Long noncoding RNA dysregulation in ischemic heart failure. J Transl Med. 2016;14:183. 27317124 10.1186/s12967-016-0926-5 PMC4912721

[22] Su Q , Zhang P , Yu D , et al. Upregulation of miR‐93 and inhibition of LIMK1 improve ventricular remodeling and alleviate cardiac dysfunction in rats with chronic heart failure by inhibiting RhoA/ROCK signaling pathway activation. Aging. 2019;11:7570‐7586. 31541994 10.18632/aging.102272 PMC6782012

[23] Li JH , Liu S , Zhou H , Qu LH , Yang JH . starBase v2.0: decoding miRNA‐ceRNA, miRNA‐ncRNA and protein‐RNA interaction networks from large‐scale CLIP‐Seq data. Nucleic Acids Res. 2014;42:D92‐D97. 24297251 10.1093/nar/gkt1248 PMC3964941

[24] Al‐Khatib SM , Stevenson WG , Ackerman MJ , et al. AHA/ACC/HRS guideline for management of patients with ventricular arrhythmias and the prevention of sudden cardiac death: executive summary: a report of the American College of Cardiology/American Heart Association Task Force on Clinical Practice Guidelines and the Heart Rhythm Society. Circulation. 2017;2018(138):e210‐e271. 10.1161/CIR.0000000000000548 29084733

[25] Liu X , Kelsoe JR , Greenwood TA . A genome‐wide association study of bipolar disorder with comorbid eating disorder replicates the SOX2‐OT region. J Affect Disord. 2016;189:141‐149. 26433762 10.1016/j.jad.2015.09.029 PMC4640946

[26] Xavier RM , Vorderstrasse A , Keefe RSE , Dungan JR . Genetic correlates of insight in schizophrenia. Schizophrenia Research. 2018;195:290‐297. 29054485 10.1016/j.schres.2017.10.021

[27] Teng Y , Kang H , Chu Y. Identification of an exosomal long noncoding RNA SOX2‐OT in plasma as a promising biomarker for lung squamous cell carcinoma. Genet Test Mol Biomarkers. 2019;23:235‐240. 30986097 10.1089/gtmb.2018.0103

[28] Li Y , Du M , Wang S , et al. Clinicopathological implication of long non‐coding RNAs SOX2 overlapping transcript and its potential target gene network in various cancers. Front Genet. 2019;10:1375. 32038720 10.3389/fgene.2019.01375 PMC6989546

[29] Feng L , Wang R , Lian M , et al. Integrated analysis of long noncoding RNA and mRNA expression profile in advanced laryngeal squamous cell carcinoma. PLOS One. 2016;11:e0169232. 28033431 10.1371/journal.pone.0169232 PMC5199101

[30] Afanas'ev I. ROS and RNS signaling in heart disorders: could antioxidant treatment be successful? Oxid Med Cell Longevity. 2011;2011:293769‐13. 10.1155/2011/293769 PMC3170796 21912722

[31] El Khoury N , Mathieu S , Fiset C. Interleukin‐1beta reduces L‐type Ca2+ current through protein kinase C activation in mouse heart. J Biol Chem. 2014;289:21896‐21908. 24936064 10.1074/jbc.M114.549642 PMC4139208

[32] Zhang Y , Liu X , Bai X , et al. Melatonin prevents endothelial cell pyroptosis via regulation of long noncoding RNA MEG3/miR‐223/NLRP3 axis. J Pineal Res. 2018;64:64. 10.1111/jpi.12449 29024030

[33] Song Y , Yang L , Guo R , Lu N , Shi Y , Wang X. Long noncoding RNA MALAT1 promotes high glucose‐induced human endothelial cells pyroptosis by affecting NLRP3 expression through competitively binding miR‐22. Biochem Biophys Res Commun. 2019;509:359‐366. 30591217 10.1016/j.bbrc.2018.12.139

[34] Song Z , Zhang Y , Gong B , Xu H , Hao Z , Liang C. Long noncoding RNA LINC00339 promotes renal tubular epithelial pyroptosis by regulating the miR‐22‐3p/NLRP3 axis in calcium oxalate‐induced kidney stone. J Cell Biochem. 2019;120:10452‐10462. 30614043 10.1002/jcb.28330

[35] Li G , Pan C , Sun J , Wan G , Sun J. lncRNA SOX2‐OT regulates laryngeal cancer cell proliferation, migration and invasion and induces apoptosis by suppressing miR‐654. Exp Ther Med. 2020;19:3316‐3324. 32266028 10.3892/etm.2020.8577 PMC7132247

[36] Wo Q , Zhang D , Hu L , et al. Long noncoding RNA SOX2‐OT facilitates prostate cancer cell proliferation and migration via miR‐369‐3p/CFL2 axis. Biochem Biophys Res Commun. 2019;520:586‐593. 31623830 10.1016/j.bbrc.2019.09.108

[37] Zhang E , Li X. LncRNA SOX2‐OT regulates proliferation and metastasis of nasopharyngeal carcinoma cells through miR‐146b‐5p/HNRNPA2B1 pathway. J Cell Biochem. 2019;120:16575‐16588. 31099048 10.1002/jcb.28917

